# Enhancing pathogen identification in patients with meningitis and a negative Gram stain using the BioFire FilmArray^®^ Meningitis/Encephalitis panel

**DOI:** 10.1186/s12941-016-0137-1

**Published:** 2016-04-21

**Authors:** Susan H. Wootton, Elizabeth Aguilera, Lucrecia Salazar, Andrew C. Hemmert, Rodrigo Hasbun

**Affiliations:** Division of Infectious Diseases, Department of Pediatrics, University of Texas (UT) Health Science Center, Houston, TX 77030 USA; Division of Infectious Diseases, Department of Internal Medicine, University of Texas (UT) Health Science Center, 6431 Fannin St. 2.112 MSB, Houston, TX 77030 USA; BioFire Diagnostics, LLC, Salt Lake City, 84108 USA

**Keywords:** Aseptic meningitis, Encephalitis, Molecular diagnostic techniques, Polymerase chain reaction

## Abstract

**Background:**

Meningitis with a negative cerebrospinal (CSF) Gram stain represents a diagnostic and therapeutic challenge. The purpose of our study was to evaluate the performance of the BioFire FilmArray^®^ Meningitis/Encephalitis (FA ME) panel in patients presenting with community-acquired meningitis with a negative Gram stain.

**Methods:**

CSF from 48 patients with community-acquired meningitis with a negative Gram stain admitted to four hospitals in Houston, TX underwent additional testing by the FA ME. FA ME results were compared to results obtained as part of routine evaluation.

**Results:**

The panel detected pathogens not previously identified in 11 (22.9 %) of 48, but did not detect pathogens identified by standard technique (West Nile virus, Histoplasma) in 5 (15.2 %) patients.

**Conclusions:**

Rapid testing for the most common pathogens causing meningitis will aid in the diagnosis and treatment of patients with meningitis.

## Background

Meningitis with a negative cerebrospinal fluid (CSF) Gram stain represents a diagnostic and therapeutic challenge, as the majority of the causative organisms are unknown [1]. Clinicians on the front lines are faced with multiple diagnostic dilemmas in an effort to ensure bacterial or other treatable infections are not missed. A variety of strategies have been employed to assist physicians including the use of advanced molecular techniques. Such advanced strategies are not widely accessible, tandem agent testing requires lengthy turn-around time at a distal reference laboratory and definitive results are complicated by specimen contamination and limited available volume [2]. Identifying the specific etiology of meningitis with a negative CSF Gram stain has important clinical, public health and antibiotic use implications. We evaluated the performance of the BioFire FilmArray Meningitis/Encephalitis (FA ME) panel in patients presenting with community-acquired meningitis with a negative Gram stain. CSF samples were obtained from patients admitted to four hospitals in Houston, TX comparing the BioFire FA to routine laboratory evaluation.

## Methods

Patients admitted between November 2008 and March 2014 with community-acquired meningitis or encephalitis (fever, headache, vomiting, photophobia, stiff neck, focal neurological symptoms), CSF cell count >5 cells/mm [3] and a negative CSF Gram stain were eligible. Residual patient CSF underwent additional testing by a research use only version of the FilmArray Meningitis/Encephalitis panel (FA ME, BioFire Diagnostics, LLC) according to the manufacturer’s instructions [3]. Briefly residual CSF sample was combined with FilmArray Sample Buffer in a 1:4 ratio and then injected into a FilmArray pouch. The panel requires 200 µL of CSF and simultaneously tests for six bacteria (*S. pneumoniae, N. meningitidis, S. agalactiae, H. influenzae, L. monocytogenes, E. coli* K1), eight viruses [Herpes simplex types (HSV) 1 and 2, Human herpesvirus 6, Cytomegalovirus, Epstein-Barr virus (EBV), Enterovirus, Parechovirus, Varicella zoster virus (VZV)] and two fungi (*Cryptococcus gattii/neoformans*). Two minutes of user hands-on time are required and comprehensive results are returned in approximately 1 h. The University of Texas Institutional Review Board approved the study and results were not used in patient management.

## Results

Of 149 patients with community-acquired meningitis, 48 (32.2 %) had residual CSF (38 adults, 10 children < age 18) available for FA ME testing. Median age of patients was 38.3 months (range 3 months–82 years) and 21 (43.7 %) were male. Twenty-five (52 %) were diagnosed with meningitis at discharge and 23 with encephalitis (47.9 %). Pathogens were identified in 14 (29.2 %) of 48 samples by routine evaluation and 15 (31.2 %) by FA ME (see Table 1). Among FA ME results, viral pathogens were most commonly detected [EBV (8), HSV2 (3), VZV (3), HSV1 (1), Enterovirus (1)], followed by bacterial [*S. pneumoniae* (2)] and fungal [*C. gattii/neoformans* (1)]. Co-detections were present in six patients (12.5 %); EBV was present in all (6) along with VZV (2), HSV1 (1), HSV2 (1), *C. gattii/neoformans* (1), and *S. pneumoniae* (1). In 11 (22.9 %) patients, FA ME identified pathogens not previously identified. Routine evaluation evaluations identified pathogens in 5 (15.2 %) of 33 FA ME negative samples [West Nile virus (WNV) (4), *Histoplasma capsulatum* (1)].

**Table 1:**
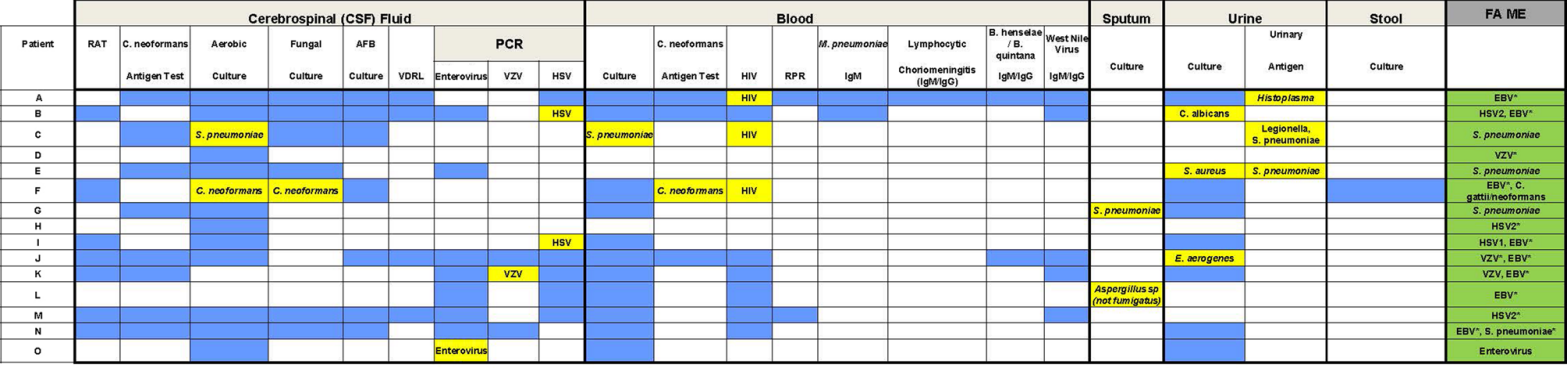
Summary of standard diagnostic testing performed in patients with community acquired meningitis with pathogens identified by FA ME (n=15)

*Blue filled cells* Test sent and negative, *yellow filled cells* Test sent and positive, *empty cells* Test not sent

*HSV* Herpes Simplex Virus, *EBV* Epstein Barr Virus, *RPR* rapid plasma reagin, *HIV* Human Immunodeficiency Virus, *VZV* Varicella Zoster Virus, *RAT* -, *VDRL* Venereal Disease Research Laboratory, *PCR* polymercase chain reaction, *AFB* Acid Fast Bacillus

* Pathogens not identified by standard evaluation (n = 11 patients)

## Discussion

Novel and fast molecular techniques are critical in identifying etiologies and starting appropriate therapy quickly in patients with meningitis. PCR assays to diagnose viral central nervous system infections have now become standard and have largely replaced viral culture [1, 4]. PCR, however, continues to be underutilized [1]. Reasons for underutilization are multiple and include variable approaches to diagnosis and variable access to such testing. The BioFire FA ME addresses such diagnostic underutilization by providing a comprehensive panel testing for 15 CNS pathogens simultaneously using a minimal amount of CSF with rapid turn-around time (1 h). Shown to be an effective alternative for other infections such as gastroenteritis [3], and bacteremia [5–8] BioFire’s FA ME was approved by the Federal Drug Administration on October 8, 2015 with promising results both in the US [9] and internationally [10, 11]. This is the second report [12] of the FA ME’s performance in the US, highlighting the importance of additional WNV and Histoplasmosis testing.

The FA ME resulted in pathogen detections not previously recognized and for which treatment is recommended [13], including VZV (n = 2), HSV (n = 2) and *S. pneumoniae* (n = 1) In one patient with VZV identified by FA ME, the routine evaluation did not include VZV testing (CSF VZV PCR or VZV IgM/IgG). Similarly, one patient with HSV identified by FA ME did not have HSV testing included in routine evaluation. In both patients, no rash was present. Appropriate laboratory studies for VZV and HSV should be ordered in any patient with unexplained meningoencephalitis, even if cutaneous manifestations are absent [14, 15]. In the patient with *S. pneumoniae* identified by FA ME but not routine evaluation, IV antibiotics were administered prior the LP, reducing the likelihood of the rapid antigen testing (RAT) and CSF culture being positive.

EBV was the most common virus identified by FA ME (n = 8), as the sole organism (n = 2) or in co-infection (n = 6). Of the patients with EBV identified by FA ME, 3 had meningitis and 5 encephalitis. The most common pathogens identified in aseptic meningitis (Enteroviruses) [1] and encephalitis (HSV, WNV and Enterovirus) [13] do not include EBV so it’s not surprising routine evaluation did not include EBV testing (EBV CSF PCR or EBV IgM, IgG) for any patient. Complications of EBV infection are diverse and include a number of neurologic manifestations such as meningitis, meningoencephalitis, cerebellitis, cranial neuritis and others. Children with EBV encephalitis rarely present with infectious mononucleosis symptoms so this particular virus should be considered in any child with acute childhood encephalitis, irrespective of associated symptoms. In a review of 21 children with EBV encephalitis, >50 % had other pathogens identified either by CSF PCR or serology. Additional reports of EBV CNS coinfections have been published [16, 17]. The role of EBV in meningoencephalitis is unclear, so it is not surprising that conventional testing did not include EBV for any patient in this study. Furthermore, the recently FDA cleared FA ME does not include EBV due to the difficulty in interpretation.

The FA ME panel was not capable of detecting organisms, such as WNV and Histoplasma, which are not included in the panel. Despite WNV being prevalent in Texas (the location of 4 enrolling hospitals) [18], only 19 (39.6 %) were tested for WNV with serology. Similarly, in a review of 323 patients with aseptic meningoencephalitis, <50 % had WNV serology performed [1]. The addition of arboviral serology to routine evaluation [1] (CSF WNV IgM, serum WNV IgM/IgG) during peak summer months would improve yield of diagnostic evaluation. Histoplasmosis is similarly common in Texas [19], often presenting in immunocompromised patients with chronic meningitis [13]. Only one patient in our study was identified to have Histoplasmosis (positive urinary *Histoplasma* antigen test) and was immunocompromised (AIDS). Special stains of sputum, blood, CSF as well as CSF antigen, CSF Ab test, and fungal culture, none of which were used, can also identify *H. capsulatum*. A fungal stain was not done in our patient, only a fungal culture.

Our study had several limitations. First, our study was observational so routine evaluation was driven by independent physicians (versus a standardized diagnostic workup). The variability in diagnostic evaluation resulted in incomplete data and hence inability to perform complete analysis of the FA ME panel dynamics including sensitivity and specificity. Secondly, the study involved 4 different institutions each with independent laboratories among which assays differing in sensitivity could have affected patient results. Furthermore, the use of residual CSF might lead to a selection bias. And finally, FA ME testing was only performed on those patients with additional CSF available, which could have resulted in a selection bias.

In conclusion, we compared the performance of standard evaluation to the BioFire Film Array Meningitis/Encephalitis panel in 48 patients. The BioFire FA ME panel detected organisms that were missed by conventional laboratory studies. The BioFire ME panel cannot replace some conventional laboratory studies, because it does not test for all organisms responsible for meningitis and encephalitis. FA ME offered comprehensive, standard, rapid testing with minimal use of CSF volume. Rapid, comprehensive testing for the most common pathogens causing meningitis will aid in the diagnosis and treatment of patients with central nervous system infections.
